# Effects of nitrogen and biochar amendment on soil methane concentration profiles and diffusion in a rice-wheat annual rotation system

**DOI:** 10.1038/srep38688

**Published:** 2016-12-08

**Authors:** Xin Xu, Zhen Wu, Yubing Dong, Ziqiang Zhou, Zhengqin Xiong

**Affiliations:** 1 Jiangsu Key Laboratory of Low Carbon Agriculture and GHGs Mitigation, College of Resources and Environmental Sciences, Nanjing Agricultural University, Nanjing 210095, China

## Abstract

The CH_4_ emissions from soil were influenced by the changeable CH_4_ concentrations and diffusions in soil profiles, but that have been subjected to nitrogen (N) and biochar amendment over seasonal and annual time frames. Accordingly, a two-year field experiment was conducted in southeastern China to determine the amendment effects on CH_4_ concentrations and diffusive effluxes as measured by a multilevel sampling probe in paddy soil during two cycles of rice-wheat rotations. The results showed that the top 7-cm soil layers were the primary CH_4_ production sites during the rice-growing seasons. This layer acted as the source of CH_4_ generation and diffusion, and the deeper soil layers and the wheat season soil acted as the sink. N fertilization significantly increased the CH_4_ concentration and diffusive effluxes in the top 7-cm layers during the 2013 and 2014 rice seasons. Following biochar amendment, the soil CH_4_ concentrations significantly decreased during the rice season in 2014, relative to the single N treatment. Moreover, 40 t ha^−1^ biochar significantly decreased the diffusive effluxes during the rice seasons in both years. Therefore, our results showed that biochar amendment is a good strategy for reducing the soil profile CH_4_ concentrations and diffusive effluxes induced by N in paddy fields.

Methane (CH_4_) is an important greenhouse gas with a global warming potential that is 34 times greater than that of the equivalent mass of carbon dioxide (CO_2_) in the atmosphere^1^. Paddy fields are considered an important source of atmospheric CH_4_. It is estimated that the CH_4_ emissions from Chinese paddy fields were 7.4 Tg yr^−1^, and they contributed to 29.9% of the global total annual emissions^2^. Rice-wheat rotation systems are ubiquitous in South and East Asia, and they play an important role in modulating the climate^3^. Fertilizer nitrogen (N) is usually required to achieve optimal yields, but when it is applied in excess, there is increased risk of pollution, such as soil acidification and increased emissions of greenhouse gas^4^,^5^, which will affect agricultural production and the ecological environment^6^,^7^. Therefore, it has become essential to explore the reasonable management steps that can be taken to achieve CH_4_ emissions mitigation without reducing crop yields from the agroecosystem by gaining a better understanding of CH_4_ production and emission processes.

Soil CH_4_ emissions depend not only on the production or oxidation rate but also on the quantity of pathways; these emissions are the result of a combination of production, oxidation and transmission^8^. The biological production of CH_4_ is mainly dominated by methanogenic archaea, and there is recent evidence for anaerobic fungi and plants that can release CH_4_^9^. However, before CH_4_ is released to the atmosphere, part of it was consumed by methanotrophs in the soil or water layer^10^. The study of CH_4_ production and distribution laws is helpful for exploring the soil-atmosphere exchange mechanism of CH_4_ in paddy soil. Yan *et al*.^11^ found that CH_4_ production in the points near the soil surface have the fastest growth rates and the shortest times to reach steady state, with the maximum concentration. Liu *et al*.^12^ reported that CH_4_ production primarily occurred in the reduced layer in a wetland, and part of the CH_4_ was oxidized when passing the oxidation zone. Yagi *et al*.^13^ analyzed Japanese rice fields and found that the top 1 cm layer always showed the highest oxidation potential in both of the plots, either in the surface layer of the paddy soil or in the rhizosphere of rice plants, and the oxidation rate in the deeper layer was nearly 58% lower than that of the surface soil. An incubation study with nine types of Philippine soil and two type of Indian soil by Mitra *et al*.^14^ revealed that the soil type has a great impact on the production potential of CH_4_ in the surface soil layer, and the topsoil was the primary source of CH_4_ in flooded rice fields, accounting for 99.9% of the total CH_4_ production; the contribution rate of the subsoil layer was only 0.05%. Kamman *et al*.^15^ analyzed the CH_4_ concentration in grassland soil profiles and showed that even in aerobic environments, there was CH_4_ production, and the reason might be related to the anaerobic micro-domain or other soil factors.

The spatial and temporal variability distribution of this gas in the soil profile will directly affect the gas exchange between the soil and the atmosphere^16^. Many studies on CH_4_ emissions have been based on the use of the closed-chamber method^15^,^17^. Using the closed-chamber method to measure CH_4_ emissions usually represents the net result of transport, consumption and production of CH_4_ in the soil^18^. Thus, the closed-chamber method applies to only the surface emission efflux, and there is a lack of research on the CH_4_ diffusion and transfer process in the soil profile. It is important to allow additional assessment of the vertical dimensions of the CH_4_ sources and sinks in the soil profile. The gradient method has already been applied to determine the soil-atmosphere exchange of CH_4_^19^. Wolf *et al*.^16^ reported that the gradient method can provide additional information about the depth profile of net gas production. Furthermore, problems associated with the use of the chambers, such as the disturbance of the concentration gradient between the soil and atmosphere and changes in the microclimate of the chamber, can be reduced or avoided^18^. Calculating the soil gas diffusive efflux using the concentration gradient method can provide a better understanding of the generation, storage, and transfer process of soil profile gas, and it can be used to estimate CH_4_ emissions at the same time^20^. There are still some limitations using the concentration gradient method that the soil was not always homogeneous and the device are limited by water flooding, even homogeneous soils can exhibit a transient soil water profile after rainfalls^21^. Previous researches were mainly concentrated on the soil CH_4_ profiles in different regions of upland soil or homogeneous soil^22^. We improved and applied the novel device to collect soil gas profiles under both drought and flooding conditions^19^.

There are a variety of impact factors on CH_4_ emissions from the soil, such as the fertilizer management^23^, field environmental factors^24^ and water regime^17^. It has been recognized that various agricultural practices including water regimes and organic matter amendments should be considered to estimate CH_4_ emission. During rice growing period, water management such as periodic drainage and intermittent irrigation can significantly reduce CH_4_ emissions^25^. Nitrogen fertilization plays an important role in CH_4_ emissions, but previous results on the effects of N fertilizers on CH_4_ emissions from rice fields are still inconsistent. Cai *et al*.^23^ proposed that a certain amount of urea could suppress CH_4_ emissions, and Wang *et al*.^5^ reported that urea can promote CH_4_ emissions. In the past few years, biochar has become an intensively discussed topic because of its proposed impacts of increased soil carbon and fertility^26^. The application of biochar can also increase plant growth by improving soil physical, chemical, and biological properties, including the soil structure, nutrient availability, and water and nutrient retention^27^,^28^. Biochar is an excellent source of organic matter to add to soil because it acts as a support material for several applications and adsorbents that remove pollutants^29^. Although comparatively few studies have addressed CH_4_ emissions after biochar addition^30^,^31^, Knoblauch *et al*.^32^ found that there was no significant difference in CH_4_ emissions between the biochar plot and the control plot during the following year, indicating that the time effect of biochar amendments needs to be further defined. Field studies of the biochar effect on the profile distribution and diffusion of CH_4_ from paddy soil is still limited. Moreover, the studies are necessary to understand the interannual variability of the combined biochar amendment and N fertilizer on the production site and diffusion of CH_4_ in the soil profile. Therefore, more studies are clearly needed to build a better understanding of biochar’s effects on the CH_4_ emissions from soil profiles with rice-wheat cropping rotations during consecutive years.

We established, a two-year field experiment from June 2013 to May 2015 with the following objectives: (1) to evaluate N fertilizer, biochar and their interaction effects on the distribution and diffusion of CH_4_ within soil profiles (2) to address the CH_4_ production location, diffusion and concentration within the soil profiles, and (3) to estimate the sources and sinks of CH_4_ in different soil depths during the rice-wheat rotations over seasonal and annual time frames from 2013 to 2015.

## Results

### CH_4_ concentrations along soil profiles in the rice-wheat rotation system

**Figure 1:**
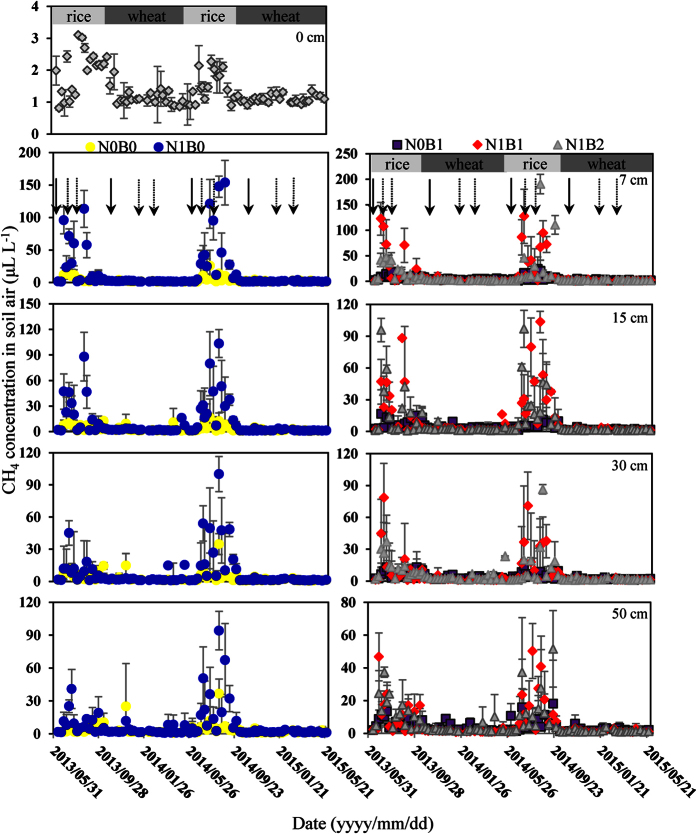
Seasonal dynamics of CH_4_ concentrations at different soil depths in the rice-wheat annual rotation system, with different N and biochar additions. The error bars show the standard deviations (n = 3). The solid and dashed arrows indicate basal fertilization and topdressing, respectively. N0B0 (no nitrogen (N) and biochar (B) amended) as control, N0B1 (only biochar amended, 20 t hm^−2^), N1B0 (only N fertilizer amended, 250 kg hm^−2^ urea), N1B1 (250 kg hm^−2^ urea and 20 t hm^−2^ biochar amended), and N1B2 (250 kg hm^−2^ urea and 40 t hm^−2^ biochar). The concentration of CH_4_ at 0 cm was used as the mean surface air sample which close to the background atmospheric value at the sampling time.

By observing the soil CH_4_ profile concentrations in different soil depths from June 2013 to May 2015 (Fig. 1), we found that the CH_4_ concentration dynamics showed a similar pattern between two rice-wheat rotations. As shown in Fig. 1, all the treatments revealed similar temporal patterns in the CH_4_ concentrations during these two years, and the profile concentrations of CH_4_ were higher during the rice seasons. However, no obvious pattern (very low concentrations; close to 0 μL L^−1^) was detected during the wheat seasons, which indicated that CH_4_ was essentially only produced and accumulated during the flooded rice seasons.

In comparison with the N0B0, the average CH_4_ concentrations from four depths in the N1B0, N1B1 and N1B2 treatments significantly increased by 261%, 205% and 165% (*P* < 0.05), respectively, especially during the second year, with increases by 413%, 285% and 205%, indicating that N fertilization significantly increased the average CH_4_ concentrations in our study (Table 1). Throughout the 2014–2015 growing season, the soil profile CH_4_ concentrations in the N1B0, N1B1 and N1B2 treatments during the rice-growing stage were significantly higher than the concentrations from 2013–2014, which suggests that there was clear inter-annual variability in the CH_4_ concentration during rice growing stages (Table 2, *P* < 0.01). Furthermore, no significant interaction effects were found between the different layers during the two different years, indicating that the soil CH_4_ concentration was consistent in the vertical distribution across the different years (Table 2).

The soil CH_4_ concentrations for the 2013 and 2014 rice seasons were both considerably influenced by N fertilizer application (Table 1, *P* < 0.05). Following basal fertilization and topdressing during the two rice seasons, the peak CH_4_ concentrations of 190.6, 103.4, 100.1 and 94.2 μL L^−1^ were measured in soil air at depths of 7, 15, 30 and 50 cm, respectively. The largest concentrations were 153.7, 127.4 and 190.6 μg N m^−2^ h^−1^ for the N1B0, N1B1 and N1B2 treatments, respectively, which occurred after the second topdressing during the rice season in 2014. Similarly, during the rice seasons of the two years (Fig. 1), the mean soil CH_4_ concentration in the four soil layers significantly increased with the N fertilizer application in comparison with the N0B0 treatment; however, the growth rate decreased with the increase in soil depth (see Supplementary Table S1, *P* < 0.05).

As shown in Table 1, no significant difference was observed in the averaged CH_4_ concentrations across all soil depths between N0B0 and N0B1, and there was also no significant difference between the different soil layers in these two treatments, indicating that no reduction effect from biochar was observed without N fertilization, with the only exception being the 50 cm layer during the rice seasons of 2013. For the rice-growing season in 2013, compared with the N1B0 treatment, the biochar amendment did not significantly affect the CH_4_ concentration in different depths of the N1B1 treatment, and all the soil CH_4_ concentrations significantly decreased in the N1B2 by 26.6% (Table 1, *P* < 0.05). However, unlike 2013, the soil CH_4_ concentrations during the rice season in 2014 were both significantly decreased in the N1B1 and N1B2 treatments by 25.0% and 40.5% compared with the N1B0 treatment (*P* < 0.05), with the only exception being the 50 cm layer, which had a similar value as the N1B0 treatment (see Supplementary Table S1). These results suggested that there was significant interaction between different treatments and years in the rice growing stages (Table 2, *P* < 0.01).

### Soil profile CH_4_ diffusive effluxes in the rice-wheat annual rotation system

**Figure 2:**
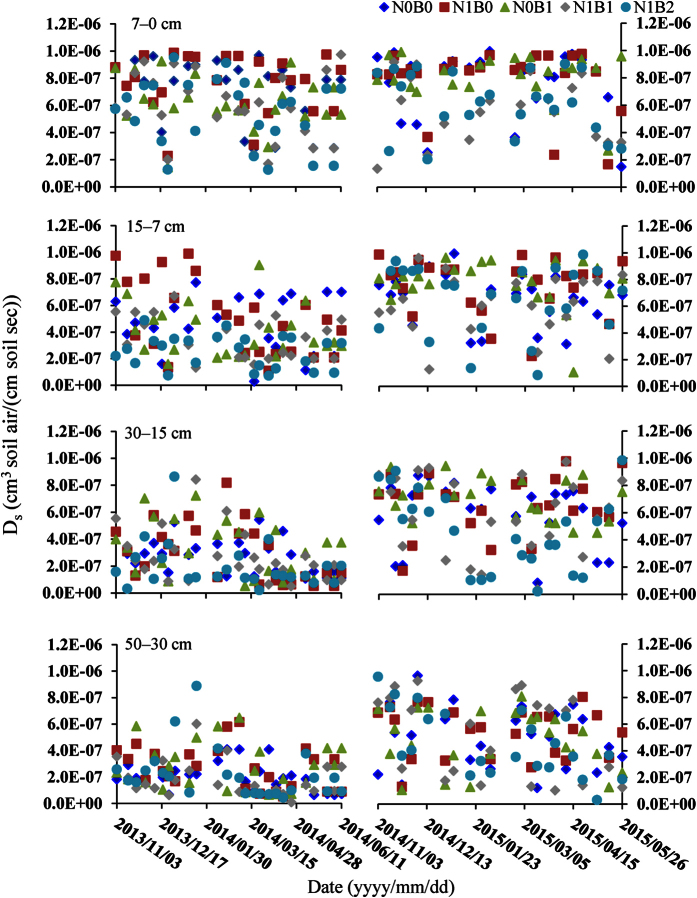
Soil gas diffusion coefficient for soil CH_4_ in different layers during the rice and wheat seasons from 2013 to 2015.

As shown in Fig. 2, the soil CH_4_ diffusion coefficient did not vary under the water-saturated condition during the flooded rice season as defined by equation (3), but it varied with the soil water-filled pore space (WFPS), which was primarily affected by precipitation over the two wheat seasons. The soil WFPS varied from 30.9% to 89.3% (see Supplementary Fig. S1). The WFPS increased in all treatments with the increase in the soil depth, but there was no significant difference observed among treatments (*P* > 0.05). The CH_4_ diffusion coefficients for the N1B0 treatment were higher than the other treatments, which decreased in the following order: N1B0 > N1B1 > N1B2 in the 7 cm depth, and it decreased with increasing depths (Fig. 2). There was no obvious rule for the five treatments in the other soil layers. Although the differences were not significant between the treatments, the differences in the CH_4_ diffusion coefficients between different soil depths were significant since both the soil moisture content and bulk density increased while the porosity decreased with depths, which all affecting the diffusion coefficient. As shown in Fig. 2, the CH_4_ diffusion coefficients were higher in 2014 to 2015 and varied from 7.80E-09 to 9.88E-07, and the coefficients from 2013 to 2014 varied from 2.04E-08 to 9.95E-07 cm^3^ soil air cm^−1^ soil^−1^ s^−1^.

The soil profile CH_4_ diffusive effluxes were calculated from the gas concentration profiles according to equations (2, 3, 4, 5). As shown in Table 3, no significant differences (*P* > 0.05) were recorded between the CH_4_ diffusive effluxes in the five treatments during the wheat seasons, and the mean values were low throughout the study. Thus, we primarily analyzed the change in CH_4_ diffusive efflux during the two rice seasons. The highest diffusive effluxes were all recorded at a depth of 7–0 cm averaged across all treatments, which showed 139.9 and 208.2 μg C m^−2^ h^−1^ during the rice seasons of 2013 and 2014, respectively. However, the diffusive effluxes were mostly negative in the deeper soil layers (Table 3). The highest diffusive influx was less than −24.8 and −52.2 μg Cm^−2^ h^−1^, which were recorded at a depth of 15–7 cm averaged across all treatments during the rice seasons of 2013 and 2014, respectively. Significant interactions were found between different depths and years of CH_4_ diffusive effluxes (Table 2, *P* < 0.001). Subsequently, the CH_4_ diffusive efflux values were found between the adjacent soil layers, which increased from positive to negative with the increase in soil depth. This finding indicated that the top 7-cm layer in rice paddy soils was the primary source of CH_4_ generation, and the deeper soil layers acted as the sink (Table 2).

**Figure 3:**
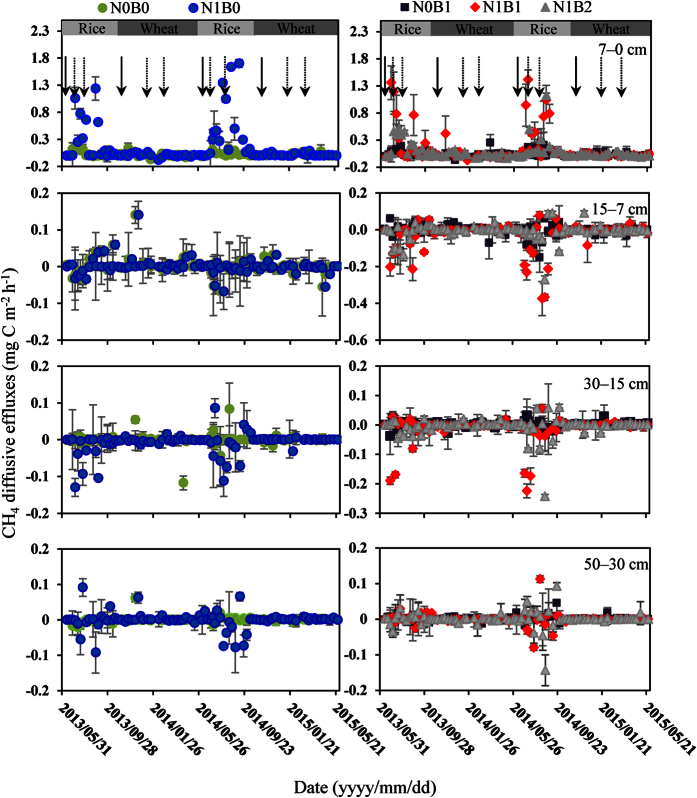
Seasonal dynamics of CH_4_ diffusive effluxes at different soil depths in the rice-wheat annual rotation system, with different N and biochar additions. The error bars show the standard deviations (n = 3). The solid and dashed arrows indicate basal fertilization and top-dressing, respectively.

The results indicated that the soil CH_4_ diffusive effluxes were significantly affected by the treatments (Table 2, *P* < 0.001). Basal fertilization and topdressing result in soil CH_4_ diffusive efflux, which increased significantly during the rice-growing stages (Fig. 3). Relative to the N0B0 treatment, the CH_4_ emission and diffusive efflux in the N1B0 treatment significantly increased from 1059.3 to 2325.4 and 7.8 to 40.6 μg C m^−2^ h^−1^ in 2013, 334.3 to 1497.9 and 9.5 to 70.2 μg C m^−2^ h^−1^ in 2014 (Table 3). However, the levels significantly decreased from 2.7 to −50.9 μg C m^−2^ h^−1^ and −7.0 to −111.5 μg C m^−2^ h^−1^ in 15–7 cm, and they displayed no obvious difference in the other layers (see Supplementary Table S2).

Biochar amendments could reduce the CH_4_ diffusive efflux that occurred from the 7 cm depth to the surface 0 cm to a certain extent, under N fertilizer application. In comparison with the N1B0 treatment, the means that averaged all depths for each treatments were decreased in N1B1 and N1B2 treatments (Table 3), the mean emission fluxes of the surface soil respectively decreased from 2325.4 to 1859.5 and 1424.2 mg C m^−2^ h^−1^ and 1497.9 to 1367.3 and 1119.9 μg C m^−2^ h^−1^ in the N1B1 and N1B2 treatment of the rice stages in 2013 and 2014, the reducing emission of CH_4_ only were significant in N1B2 treatments. Be consisted with the result of the mean emission fluxes, the mean soil CH_4_ diffusive effluxes to a depth of 7 cm were decreased by 2.95% and 38.82% in the N1B1 treatment, and they significantly decreased by 27.80% and 51.64% in the N1B2 (*P* < 0.05) of the rice seasons in 2013 and 2014, respectively (see Supplementary Table S2). By contrast, there was no difference between the CH_4_ emission and diffusive efflux of the N0B1 and N0B0 treatments, indicating that the biochar amendment had no effect on the CH_4_ diffusion without the N fertilizer application in the annual rice-wheat rotations. Similarly, in 2013 and 2014, there was no obviously difference between the N1B0 and N1B1 treatments, as shown in Table 3. Thus, we can deduce that the amendment of 20 t ha^−1^ biochar with N fertilization has no significant effect on reducing the diffusive effluxes of CH_4_ in the soil profile.

### Comparative study of soil CH_4_ emission and diffusion efflux

The top 7-cm soil depth performed as the main source for the CH_4_ production while the diffusion effluxes in other layers all were low or negative (Tables 1 and 3). Thus the CH_4_ diffusion in the 7–0 cm soil depth was selected to compare with the CH_4_ emissions of soil surface.

Similar pattern were shown in 2013–2014 and 2014–2015, that the emission fluxes were nearly 2.5 to 38.8 times significantly higher than the diffusion effluxes (Table 3, *P* < 0.05). Regression analysis results showed that under the condition of conventional N fertilization, there was a significant positive correlation between the CH_4_ diffusive effluxes in the 7–0 cm soil layer and the CH_4_ emission fluxes. While the diffusive effluxes in the deeper soil layers showed significant negative correlation with the emission efflux (Table 4, *P* < 0.05). The above results indicated that the top 7-cm layer in rice paddy soils was the primary source of CH_4_ production and oxidation, while the deeper soil layers performed as the sink. Although there obtained the certain correlation between the CH_4_ diffusive effluxes between the CH_4_ emission fluxes, but there were significant differences between the values of them (Table 3 and see Supplementary Table S2).

## Discussion

The soil CH_4_ concentrations were considerably influenced by N applications that resulted in peaks throughout the soil profile (Fig. 1), and the concentrations performed higher in the upper (7 cm and 15 cm) layers than in the deeper ones (30 cm and 50 cm) during the rice seasons (Table 1). The probable reason might be the absence of substantial amounts of methanogens in the deeper soil layers^33^. Conrad *et al*.^34^ explained that the decrease in CH_4_ below the oxic surface layers was likely related to the presence of rice roots in these soil layers, which provided a favorable environment for methanotrophs and allowed for the release of CH_4_ by plant vascular transport. In our study, significant interactions between the treatments and years were only found in the soil CH_4_ concentrations profiles during the rice growing stages (Table 2, *P* < 0.05). Given that the surface water was maintained continuously by irrigation at the rice-growing stage, the water regime in the field was not considered to influence the yearly variation in the CH_4_ emission^35^. The microbial and root metabolic activity were inhibited under the low soil water content during the wheat season, while soil oxygen would be depleted when pore spaces were saturated with water under the very high soil water content^36^, that can reduce the oxidation of CH_4_. Watanabe *et al*.^24^ explained that the temperature is considered the most influential factor when the fertilizer application and other cultivation methods are constant. There was only a small amount of difference between the soil temperatures from 2013–2014 and 2014–2015. Thus, we further hypothesized that the higher concentrations of CH_4_ in 2014 might be related to the fact that the surface soil contained more organic material and microorganisms following more cultivation and agricultural management^36^,^37^.

The nitrogen application significantly increased the CH_4_ concentrations from all the soil layers in our study (Table 1, *P* < 0.01), primarily because the N fertilizer application can stimulate CH_4_ production by increasing the growth of rice plants and root exudation, which can promote the carbon supply for methanogens^38^ in soil layers to provide an enabling environment for CH_4_ generation. Conrad *et al*.^10^ proposed that a favorable habitat for the growth of methanogens and sufficient substrates are prerequisites for CH_4_ generation. Wang *et al*.^5^ also showed that the observed increase in CH_4_ production might be related to the fact that N fertilizer can decrease the soil C:N ratio and promote the activity of soil microorganisms. The increased CH_4_ concentrations associated with N fertilization are likely ascribed to the fertilizer-induced reduction in soil CH_4_ oxidation in different soil layers^39^. As a result of the increase in the soil NH_4_^+^-N concentration after the application of N fertilizer, because of the competition effect, a higher concentration of NH_4_^+^-N may reduce the probability of CH_4_ oxidization by methanotrophs^40^. Mohanty *et al*.^41^ hypothesized that NH_4_^+^-N is a competitive inhibitor of CH_4_ monoxygenase. The research of Wu *et al*.^42^ demonstrated that N fertilizers can directly alleviate N limitation to methanogens especially near the rhizosphere where concentration of ammonia is lower due to rice plants uptake.

Biochar amendments of 20 t ha^−1^ and 40 t ha^−1^ with N fertilization reduced the soil CH_4_ concentration during the rice-wheat rotation systems from 2013 to 2015 (Table 1, Fig. 1, *P* < 0.05), significantly at the 0–15 cm depth (see Supplementary Table S1, *P* < 0.05), which is consistent with the study by Scheer *et al*.^31^ in which the biochar amendment can reduce the generation and emission of soil CH_4_. Han *et al*.^43^ even found that there was an increase in CH_4_ uptake in soil amended with biochar. However, elevated CH_4_ production and emission following biochar amendments in soil have been shown in previous studies^30^,^32^. The contrasting observations in our study may be explained by the different soil types and site conditions as well as the properties of biochar having a different effect on the CH_4_ emissions of soil. According to Van Zwieten *et al*.^44^, the labile organic C added by the biochar can provide abundant available substrates for methanogens and create locally anaerobic microsites in soil that favors CH_4_ production. In addition, biochar can increase the air permeability of soil with a greater specific surface area^27^, which was more beneficial to the growth of the methanotrophs. Feng *et al*.^45^ found that biochar can increase the abundance of methanotrophs in the paddy soil and reduce the ratios of methanogens to methanotrophs. While Han *et al*.^43^ reported that the decreased CH_4_ release was primarily attributable to the decreased activity of methanogens along with the increased CH_4_ oxidation activity, Silber *et al*.^46^ showed that biochar can also increase the soil cation exchange capacity (CEC) to improve the soil pH, which can further inhibit the activity of methanogens. Yu *et al*.^47^ reported that soil moisture can affect CH_4_ emissions by directly influencing methanogenic and methanotrophic activities and by indirect effects through changes in soil aeration and redox potential. The CH_4_ uptake appeared to be very sensitive to the soil moisture content at all depths^48^ since soil water content and soil electrical conductivity (EC) were the most influential factors driving the changes in the microbial community during agricultural practices^38^. These findings indicated that the different effects of biochar on the CH_4_ concentration in the treatment amended with biochar between different seasons and different layers in our study may be related to the different moisture levels.

In this study, the application of N fertilizer with biochar had no significant effect on the soil CH_4_ concentration during the wheat seasons (Table 1), which might be related to the variety of water contents present during the rice and wheat growing seasons. The environment factors might reduce the biochar effect to some extent.

The emission of CH_4_ is the combined consequence of soil production, oxidation and diffusion in soil^8^, and the difference in the CH_4_ concentrations in different soil profile depths is the primary dynamic mechanism of CH_4_ emission^33^. The results of the soil CH_4_ concentration and diffusive flux (Tables 1 and 3 and Figs 1 and 3) can directly reflect the generation and storage site of CH_4_ in soil, providing detailed information for research on the source and sink of CH_4_ in paddy soil.

Because of the high spatial variation in CH_4_ concentrations, there is a spatial variation in the soil CH_4_ diffusive efflux in the field under flooded conditions during the rice seasons. As previously described, the soil CH_4_ concentration patterns varied with the rice and wheat seasons (Fig. 1 and Table 1), affecting the concentration gradient of CH_4_ in the soil profile, which might be explained by the fact that the soil was under the flooded anaerobic conditions that prevailed in the rice paddy, and CH_4_ was produced when the organic materials decomposed under oxygen-deprived conditions^49^. A large portion of the CH_4_ generated in the soil profile might be oxidized, and it is estimated that more than 50–90% of the CH_4_ produced belowground is oxidized before reaching the atmosphere^34^.

The distribution of the soil CH_4_ concentration and diffusion showed the same dynamic rule during the rice-wheat crop rotations from 2013–2015. As previously described, with only the exception of the soil CH_4_ diffusive efflux at 7–0 cm during the two rice growing stages, the values of the soil CH_4_ diffusive efflux during the wheat seasons and the rest of the soil depths were small or negative, and the average soil CH_4_ concentrations and diffusive effluxes from the 7–0 cm depth were significantly higher than that of the subsoil (Tables 1, 3 and Tables S1 and S2). As a complicated heterogeneous system, the different soil layers have different structural features, and there are differences in their contents of soil organic matter^37^. With a higher soil organic matter content, the total N and NH_4_^+^-N was greater in the soil surface layer than in the deeper depths^50^,^51^, and the CH_4_ concentration was significantly promoted in the surface layer of the paddy field, making the surface soil the primary CH_4_ generation area. Hütsch *et al*.^40^ reported that the topsoil was more conducive to the generation of CH_4_, because even in an anaerobic state, the lower soil layers had fewer nutrient substrates; thus, the CH_4_ content might also be relatively lower than that of the topsoil. The vertical distribution of CH_4_ storage in the soil profile also reflects the distribution rule of soil organic matter decomposition and CH_4_ diffusion in the soil profile. The significant interaction of the soil CH_4_ diffusive effluxes between different depths and years (Table 2, *P* < 0.001) may be related to the different gas diffusion coefficients (*Ds*) and the soil WFPS in the different depths. The study by Pingintha *et al*.^52^ explained that with the lower suction of soil water in the surface soil than in lower depths for agricultural soil, the *D*_*s*_ in the soil decreased with the increase in depth, and thus the gas in the surface soil is more prone to being diffused to the atmosphere, as opposed to the other layers. The CH_4_ diffusive efflux in shallow soil can directly reflect the CH_4_^53^ emissions. Similar to the grassland study of Hartmann *et al*.^39^, we observed that the soil CH_4_ concentrations were always decreased with the soil depth, which indicated that the depths below 7 cm in the soil and the soil in the wheat seasons were the net sink for atmospheric CH_4_ and primarily acted in the absorption of CH_4_ (Table 3). The average CH_4_ concentrations increased with the N fertilization application in 2014–2015, which was more obvious than that in 2013–2014. Compared with the N0B0 treatments with less available nutrient substrates, the treatments received the N fertilization and biochar amendment showed more obvious effects in the second year than the first year. The fact that N fertilization would promote the growth of plant^38^ and then provide the substrates to methanogens^34^ and play an important role in the transport of CH_4_^8^ may explain this phenomenon.

High soil CH_4_ concentrations lead to more accumulation and emissions^19^. However, in comparison with the field experiments that monitored the CH_4_ emissions from the same plots in Li *et al*.^54^, the emission effluxes of CH_4_ were clearly higher than the diffusive effluxes that we observed. Although there was a significantly positive correlation between the soil CH_4_ emission fluxes and diffusive effluxes in 7–0 cm in the treatments with N fertilizer application in rice-wheat crop rotations of 2013–2015, there were no significantly positive correlation in the treatments without N fertilization, nor the other soil layers (Table 4) associated with the low ratios between CH_4_ diffusion efflux and surface emissions (Table 3). Nitrogen fertilization amendment could significantly increase the production of CH_4_, correspondingly increased the CH_4_ diffusion and emission as previously mentioned.

The CH_4_ diffusive effluxes in the top 7-cm soil layers were significantly lower than the emissions of the surface soil in rice-wheat rotation systems, the values of the CH_4_ diffusive effluxes in 7–0 cm soil depth being only 2.5 to 28.6% of the surface soil emissions (see Supplementary Table S2). The reason might be due to the facts that the soil CH_4_ is mainly transported by plants or bubbles other than free diffusion into the atmosphere during rice season with saturated soil moisture condition, and agreed well that the concentration gradient method produced smaller estimates^18^. While during the wheat season the diffusion was a major transport way of soil gas when the soil moisture contents were low, soil CH_4_ might easily be oxidized in the aerobic zone of surface layer^8^. In addition, the position deviation of the observation and the uneven distribution of soil moisture and organic matter were also the possible causes for the difference between surface emission and diffusion^21^. Similarly, Hendriks *et al*.^55^ reported soil CH_4_ diffusion rates were lower than the CH_4_ emissions observed by the chamber method in similar paddy fields. The transportation of CH_4_ emissions from paddy soil into atmosphere is the comprehensive result of production, oxidation and diffusion, while only calculating the CH_4_ diffusion by concentration gradient method to estimate the CH_4_ emissions will get the relatively smaller values. However, Dunfield *et al*.^22^ found a good correlation between the diffusion calculated by the concentration gradient and the emission measured by the chamber method due to the homogeneous unstructured soil conditions. Therefore use the concentration gradient method to estimate the amount of CH_4_ diffusion in rice−wheat rotation system cannot completely take place of the observation on soil surface emissions, which needs a further research.

## Materials and Methods

### Field site and experimental design

A field experiment that involved the monitoring of the emission and diffusion of CH_4_ in the soil profile was performed in a typical rice paddy in MoLing Town (31°58′ N, 118°48′ E), Nanjing, Jiangsu Province, China. The field was cultivated under a crop rotation system of rice in summer (June to October) and wheat in winter (November to May). The site is characterized by a subtropical humid monsoon climate with a mean annual air temperature of 15.7 and 16.9 °C and precipitation of 1,050.2 and 1,072.4 mm for two years. The field soil is classified as *Irragric Anthrosols*^56^ with a silty clay loam texture consisting of 14% clay, 6% sand, and 80% silt. The physicochemical properties of the soil in the 0–50 cm horizon are shown in Supplementary Table S3. The soil pH was higher in the deeper (7.01 ± 0.15 in 15–30 cm and 6.72 ± 0.20 in 30–50 cm) layers than in the upper ones (5.91 ± 0.16 in 0–7 cm and 6.53 ± 0.12 in 7–15 cm); and the surface soil layers have the highest organic carbon, 16.23 ± 0.83 g C kg^−1^; total N, 1.43 ± 0.07 g kg^−1^; and CEC (cation exchange capacity), 28.60 ± 0.11 cmol kg^−1^, while the soil bulk density increased with the soil depth increased, being 1.41 ± 0.01 g cm^−3^ in the 30–50 cm soil layers. The daily mean air temperatures and precipitation during the study period from June 19, 2013, to May 31, 2015, are given in Supplementary Fig. S2.

Five treatments were established in three replicates in a completely random design as follows: N0B0, N0B1, N1B0, N1B1, and N1B2. The same 15 field plots were used for all the two rice-wheat rotations in both years. In brief, biochar was added to the soil at a rate of 0 (as the control), 20 and 40 t ha^−1^ (which were coded as B0, B1, and B2, respectively), and N fertilizer (urea) was applied at two rates of 0 (N0) and 250 kg N ha^−1^ crop^−1^ (N1). Each plot had an area of 5 m × 4 m. In each treatment, the biochar, which was originally in particulate form, was added once to the paddy fields in June 2012 and was incorporated into the soil by plowing to a depth of 50 cm. The biochar used in this experiment was produced from wheat straw at a temperature of approximately 350–550 °C by a local pyrolysis plant in a vertical kiln constructed from refractory bricks at Sanli New Energy Company, Henan, China. The biochar had a total C content of 467 g kg^−1^, a total N content of 5.6 g kg^−1^, a pH of 9.4 (1:1.25 H_2_O), an ash content of 208 g kg^−1^ and a cation exchange capacity of 24.1 cmol kg^−1^.

### Field plot management

The field management including the crop species, fertilizer application rates and methods, tillage, irrigation, pesticide and weed control were performed in accordance with local practices (Supplementary Table S4). No irrigation was performed during the wheat season. In treatments receiving N fertilization, urea was applied at a rate of 250 kg N ha^−1^ and split into a 4:3:3 ratio of basal fertilizer and two topdressings for both the rice and wheat crops. The topdressing was applied at the tillering and panicle stages of the rice crop and at the seedling establishment and elongation stages of the wheat crop. Both calcium superphosphate and potassium chloride were applied as basal fertilizers at rates of 60 kg P_2_O_5_ ha^−1^ crop^−1^ and 120 kg K_2_O ha^−1^ crop^−1^ during both the rice and wheat seasons. Rice was transplanted on June 19, 2013 and June 10, 2014 and harvested on October 18, 2013 and October 25, 2014, respectively. Winter wheat was directly sown on November 3, 2013 and November 13, 2014 and then harvested on May 28, 2014 and May 26, 2015, respectively.

### Gas sample collection and measurement

Observations of the CH_4_ soil profile were conducted during the rice-wheat growing season from June 13, 2013, to May 31, 2015. The monitoring of CH_4_ emission fluxes were performed using a static chamber method and we constructed soil gas collection tubes to obtain samples from different soil depths (the samples were centered at 0, 7, 15, 30 and 50 cm) at a single site^19^. There was one sampling column collecting soil gas at different depths at the same position and one sampling chamber on the ground in each plot. The 0–15 cm soil layer was the plough layer, and the redox mainly happened in this layer; 15–30 cm soil layer was the plow pan; 30–50 cm soil layer was the saturated soil layer. The gas sampler was installed along the same vertical section of the soil profile. Every sampler consists of four independent chambers, which represent the CH_4_ concentrations at depths of 7, 15, 30, and 50 cm (see Supplementary Fig. S3). The internal headspace volume for each sampling unit was approximately 48 cm^3^. The structure of the samplers consisted of long, 50-cm polyvinyl chloride (PVC) tubes with four individual units (the inside diameter was 4.0 cm and the height was 5.0 cm) connected together, and each unit contained eight uniformly distributed holes that were 1.2 cm in diameter each and covered by two layers of 80-mesh nylon for soil gas equilibration from the surrounding soil layers. For each gas equilibration unit, silicone tubing (inner diameter = 4.76 mm; outer diameter = 7.62 mm) was fixed inside the sampler and penetrated out of the PVC tube surface. The ends of the four stretching silicone tubes were fitted with three-way stopcocks that allowed the sampling of the subsurface gases above the soil surface. A PVC plate was installed between each individual unit to ensure isolation. Soil gas samples were collected by first drawing 5 mL of gas to purge the volume of the spaghetti tubing and then drawing a 20 mL sample using a syringe. The valves were kept closed between samplings to ensure that subsurface gas samplers were not contaminated with atmospheric air. The CH_4_ sampling tubes were left in place for the whole two years.

The gas samples were analyzed for CH_4_ concentrations using a gas chromatograph (Agilent 7890 A Shanghai, China) equipped with a hydrogen flame ionization detector (FID). The carrier gas was nitrogen, and it had a flow rate of 40 mL min^−1^. The temperatures of the oven and the FID were 50 and 300 °C, respectively. The mean concentrations and soil diffusive fluxes of CH_4_ for the rice and wheat were all calculated as the average of all the measured concentrations, and fluxes that were weighted by the two measurements at each sampling point divided by the time interval. The CH_4_ fluxes were calculated using the linear increases in gas concentration with time. The concentration of CH_4_ at 0 cm was used as the mean surface air sample, which close to the background atmospheric value at the sampling time (Fig. 1), sampled from the surface ground in each plot.

All analyses of soil chemical properties were based on the standard methods for soil analyses described by Sparks *et al*.^57^. We monitored only the soil moisture contents at depths of 7, 15, 30, and 50 cm during the wheat season because the soil moisture content did not vary during the flooded rice season under the water-saturated condition. The water content was converted to water-filled pore space (WFPS) with the following equation^58^:


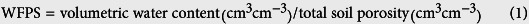


here, the total soil porosity = [1− (soil bulk density (g cm^−3^)/2.65)] with an assumed soil particle density of 2.65 (g cm^−3^). The soil bulk density at different depths was determined using the cutting ring method^59^.

### Data calculation

Soil CH_4_ concentration data were used to estimate the diffusive efflux of CH_4_ within different soil depths with Fick’s Law as follows:


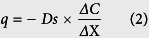


where *q* is the diffusive efflux of CH_4_ (ng cm^−2^ s^−1^), *D*_*s*_ is the soil gas diffusion coefficient of CH_4_ in the soil (cm^3^ soil air cm^−1^ soil s^−1^), *C* is the concentration of CH_4_ (ng m^−3^), *ΔC* is the concentration difference between two depths, *X* is the vertical position (cm) (i.e., 0, −7, −15, −30 and −50 cm according to the soil stratification and redox conditions in the paddy field), *ΔX* is the difference in depths between two adjacent soil layers, and *ΔC/ΔX* is the vertical soil CH_4_ gradient (ng cm^−3^ cm^−1^). The CH_4_ diffusive efflux is the rate of CH_4_ efflux from the lower designated soil layer to the upper soil layer (i.e., 7–0, 15–7, 30–15 and 50–30 cm). A positive value for the diffusive efflux represents CH_4_ efflux, which indicates CH_4_ diffusing to the upper soil layer, and negative values represent CH_4_ diffusing to the lower soil layer, as expressed as the CH_4_ diffusive influx. The effective diffusion coefficient *D*_*s*_ of CH_4_ in the soil is lower than that in the atmosphere and can be expressed as follows:


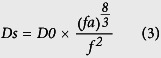



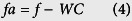



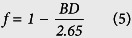


where *D*_*0*_ is the diffusion coefficient of the atmosphere (cm^2^ s^−1^) and is determined as 0.156 cm^2^ s^−1^ for CH_4_ at T = 293.2 K and P = 101.3 kPa^60^. The *f*_*a*_ and *f* are the air-filled porosity (cm^3^ cm^−3^) and total porosity of the soil (cm^3^ cm^−3^), respectively. *WC* is the soil volumetric moisture content (cm^3^ cm^−3^), and *BD* is the soil bulk density (g cm^−3^), which was assumed to be constant for each corresponding soil layer, although some variations may have occurred during crop rotation. Depending on the gas and chemistry of the soil solution, the amount of gas stored in the aqueous phase may be neglected. Although there may have been some variations in the actual bulk density among the replications, treatments and crops rotations (±0.1 g cm^−3^), they were not expected to be significant relative to the changes in CH_4_ diffusive efflux.

### Statistical analysis

To examine differences in the mean soil CH_4_ concentrations and diffusive effluxes data among different treatments, years, depths were subjected to linear mixed-effects models (LMMs). The data for the wheat and rice were analyzed separately. Models included treatment (N0B0, N0B1, N1B0, N1B1, N1B2), year (2013–2014, or 2014–2015) and depth (0–7, 7–15, 15–30, 30–50 cm) as fixed effects and plot was included as a random effect. The depth was treated as a multilevel factor. We validated the use of LMMs with restricted maximum likelihood estimation method (REML) based on the normalized scores of standardized residual deviance of response variables for the soil CH_4_ concentrations and diffusive effluxes. Statistical analyses were performed with the statistical package R 3.0.0 (using the ‘lme4’ package) with a significance level of alpha = 0.05 for LMMs (R Development Core Team 2013).

Before the analysis, the data from each depth interval were averaged within each ring, the ring refers to the individual units (the inside diameter was 4.0 cm and the height was 5.0 cm, see Supplementary Fig. S3) and each ring was treated as a statistical replicate for the treatments. Simple and multiple linear and nonlinear regression analyses were used to examine the relationships between the CH_4_ fluxes measured using the static chamber and gas concentration gradient methods. The multiple comparisons among the means were done based on the pooled errors from the analyses summarised in Table 2, performed with the statistical package R 3.0.0 (using the ‘lme4’ package). We define an emission peak as a peak that was significantly higher than the previous and following effluxes. Normal distribution and variance uniformity were checked, and all the data were consistent with the variance uniformity (*P* > 0.05) within each group. The results are presented as the means and standard deviation (mean ± SD, n = 3).

## Additional Information

**How to cite this article**: Xu, X. *et al*. Effects of nitrogen and biochar amendment on soil methane concentration profiles and diffusion in a rice-wheat annual rotation system. *Sci. Rep.* **6**, 38688; doi: 10.1038/srep38688 (2016).

**Publisher's note:** Springer Nature remains neutral with regard to jurisdictional claims in published maps and institutional affiliations.

## Supplementary Material

Supplemantary Material

## Figures and Tables

**Table 1:** Soil CH_4_ concentration profiles (unit) across all treatments and all depths over 2013–2015 growing seasons.

	CH_4_ concentration (μL L^−1^)			
	Rice season		Wheat season	
	2013–2014	2014–2015	2013–2014	2014–2015
Treatment				
N0B0	5.13 ± 0.82*,§	4.85 ± 0.57*,§	2.16 ± 0.50	1.47 ± 0.10
N1B0	18.53 ± 1.91	24.87 ± 3.35	2.02 ± 0.45	1.55 ± 0.18
N0B1	6.90 ± 0.51	5.32 ± 0.89	2.46 ± 0.19	1.70 ± 0.15
N1B1	15.66 ± 3.38	18.65 ± 2.46	1.74 ± 0.25	1.39 ± 0.03
N1B2	13.60 ± 1.32	14.81 ± 3.94	2.12 ± 0.19	1.53 ± 0.13
Soil depth				
7 cm	16.65 ± 2.84¶	19.97 ± 5.56¶	1.81 ± 0.27¶	1.55 ± 0.14
15 cm	12.80 ± 1.17	13.13 ± 2.94	2.16 ± 0.22	1.46 ± 0.07
30 cm	9.50 ± 1.78	12.18 ± 3.46	2.12 ± 0.35	1.50 ± 0.13
50 cm	8.91 ± 1.02	9.51 ± 3.74	2.32 ± 0.55	1.61 ± 0.24

B0, B1 and B2 represent biochar applied at the rates of 0, 20 and 40 t ha^−1^, respectively; N0 and N1 represent N fertilizer applied at the rates of 0 and 250 kg N ha^−1^ crop^−1^, respectively.

Data are Means ± SD.

^*^Indicates significant interaction between treatment and soil depth.

^§^Indicates significant difference among treatments across all depths.

^¶^Indicates significant difference among soil depths across all treatments.

**Table 2:** Results of linear mixed effect models for wheat and rice seasons with the treatments (T), depths (D), and years (Y) as fixed effects and plot as random effects.

Crop season	Response variable	CH_4_ concentration (μL L^−1^)			CH_4_ diffusive efflux (mg C m^−2^ h^−1^)		
		*df*	*F-value*	*P-value*	*df*	*F-value*	*P-value*
Wheat season	T	4	2.885	0.079	4	0.594	0.668
	D	3	2.463	0.070	3	42.298	0.000
	Y	1	59.017	0.000	1	0.045	0.833
	T × D	12	1.322	0.226	12	2.126	0.024
	T × Y	4	0.973	0.428	4	0.444	0.777
	D × Y	3	2.026	0.118	3	2.450	0.070
	T × D × Y	12	0.616	0.822	12	1.214	0.289
Rice season	T	4	41.148	0.000	4	9.023	0.000
	D	3	37.013	0.000	3	255.451	0.000
	Y	1	7.104	0.009	1	2.941	0.090
	T × D	12	7.704	0.000	12	30.069	0.000
	T × Y	4	4.494	0.003	4	0.844	0.501
	D × Y	3	1.315	0.276	3	11.577	0.000
	T × D × Y	12	0.637	0.804	12	2.647	0.005

**Table 3:** The diffusive effluxes and surface emission fluxes of CH_4_ (unit) across all treatments and all depths over 2013–2015 growing seasons.

Treatment	CH_4_ fluxes (μg C m^−2^h^−1^)							
	Rice season				Wheat season			
	2013–2014		2014–2015		2013–2014		2014–2015	
	Means	Surface emission fluxes	Means	Surface emission fluxes	Means	Surface emission fluxes	Means	Surface emission fluxes
N0B0	7.8 ± 16.7*,§	1059.3 ± 36.4	9.5 ± 21.4*,§	334.3 ± 49.9	4.4 ± 6.9*	159.3 ± 12.3	4.0 ± 8.8	141.2 ± 46.8
N1B0	40.6 ± 132.4	1056.6 ± 31.2	70.2 ± 242.4	553.8 ± 138.4	3.8 ± 3.9	160.4 ± 17.1	6.4 ± 8.6	160.2 ± 66.3
N0B1	13.3 ± 27.8	2325.4 ± 69.7	11.4 ± 30.9	1497.9 ± 68.1	7.3 ± 14.6	176.8 ± 13.9	5.6 ± 11.5	120.0 ± 23.5
N1B1	38.0 ± 130.5	1859.5 ± 70.5	52.6 ± 173.2	1367.3 ± 83.0	3.8 ± 8.8	173.2 ± 10.1	3.3 ± 8.2	146.5 ± 13.3
N1B2	29.6 ± 77.7	1424.2 ± 27.1	37.4 ± 226.4	1119.9 ± 63.3	4.1 ± 8.0	143.3 ± 16.2	5.8 ± 12.6	109.6 ± 90.4
Soil depth	Means							
0–7 cm	139.9 ± 95.4¶		208.2 ± 165.2¶		14.5 ± 9.9¶		19.6 ± 3.8¶	
7–15 cm	−24.8 ± 29.0		−52.2 ± 43.5		4.6 ± 6.1		−0.3 ± 4.2	
15–30 cm	−11.2 ± 9.8		−3.5 ± 11.9		−0.4 ± 1.9		0.1 ± 1.8	
30–50 cm	−0.6 ± 2.0		−7.5 ± 11.0		0.1 ± 1.8		0.7 ± 0.9	

B0, B1 and B2 represent biochar applied at the rates of 0, 20 and 40 t ha^−1^, respectively; N0 and N1 represent N fertilizer applied at the rates of 0 and 250 kg N ha^−1^ crop^−1^, respectively.

Data are Means ± SD.

^*^Indicates significant interaction between treatment and soil depth.

^§^Indicates significant difference among treatments across all depths.

^¶^Indicates significant difference among soil depths across all treatments.

**Table 4:** Correlation coefficients between CH_4_ emissions and diffusive effluxes within the soil profiles among the different treatments from the rice-wheat annual rotations.

Year	Diffusive flux	Treatment				
		N0B0	N0B1	N1B0	N1B1	N1B2
2013–2014	7–0	0.207	0.048	0.299*	0.347*	0.501**
	15–7	−0.151	0.209	0.144	−0.404**	−0.519**
	30–15	−0.050	0.244	0.050	−0.111	−0.141
	50–30	−0.318*	0.110	−0.053	−0.353*	−0.119
2014–2015	7–0	0.124	0.228	0.291*	0.416**	0.404**
	15–7	−0.111	−0.093	−0.145	−0.506**	−0.346*
	30–15	0.105	−0.049	0.117	0.040	−0.111
	50–30	0.133	−0.116	−0.085	−0.178	−0.072

^*^P < 0.05, ^**^P < 0.01, ns not significant.
