# Supplementary material for: Effects of nitrogen and biochar amendment on soil methane concentration profiles and diffusion in a rice-wheat annual rotation system
Source: Sci Rep. 2016 Dec 8;6:38688. doi: 10.1038/srep38688 (PMC5144129; doi:10.1038/srep38688)
Supplement: Supplemantary Material [file srep38688-s1.pdf]

Supplementary material

Effects of nitrogen and biochar amendment on soil methane concentration profiles and diffusion in a rice-wheat annual rotation system

Xin Xu, Zhen Wu, Yubing Dong, Ziqiang Zhou, Zhengqin Xiong

Figure legends

Fig. S1. Dynamics of soil water-filled pore space (WFPS) of the two wheat seasons during 2013–2015. The error bars show the standard deviations (n=3).

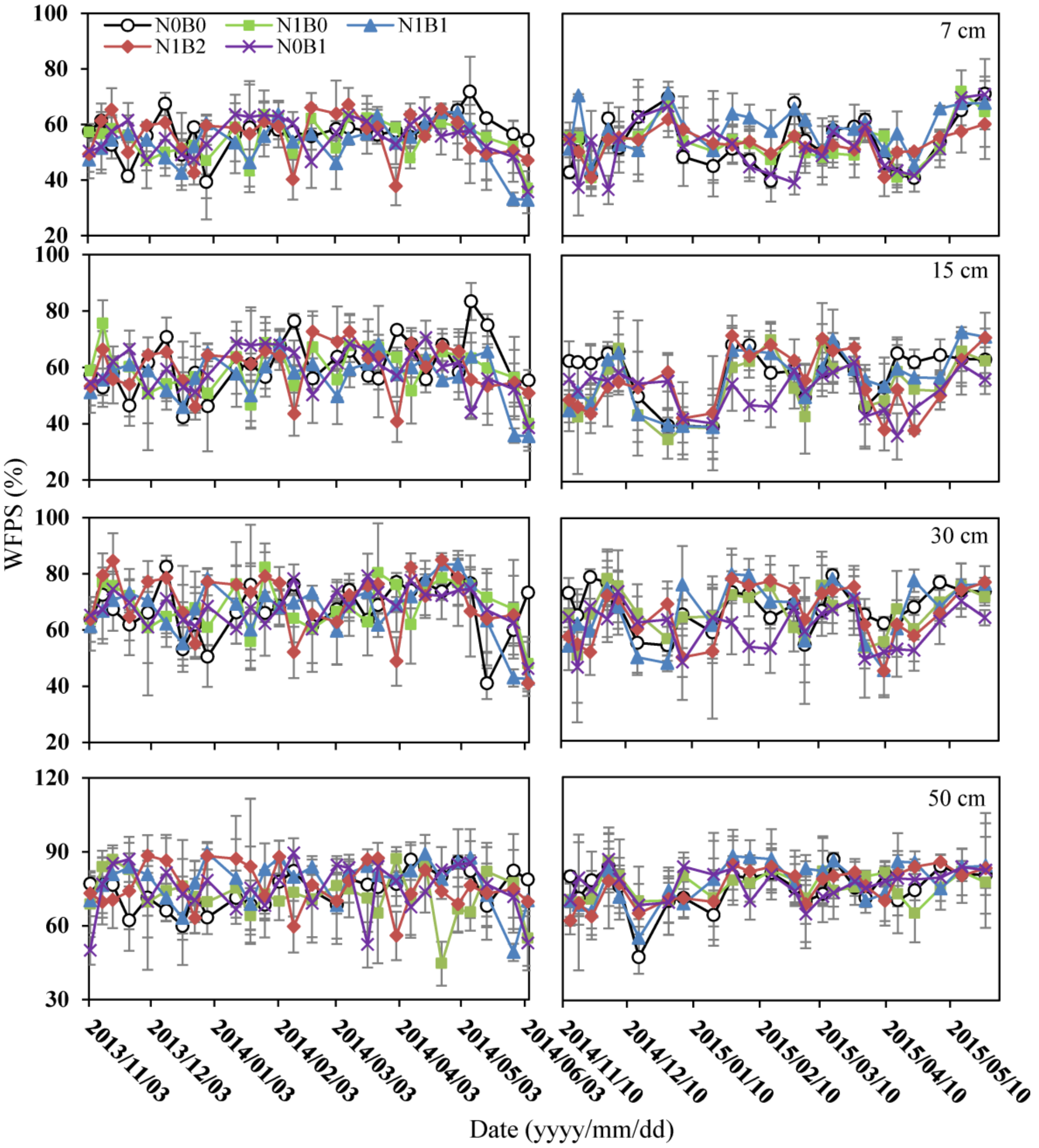

Fig. S2. Daily mean air and soil temperature (℃) and precipitation (mm) during the rice-wheat rotation in 2013-2015 in MoLing Town, Nanjing, Jiangsu Province, China.

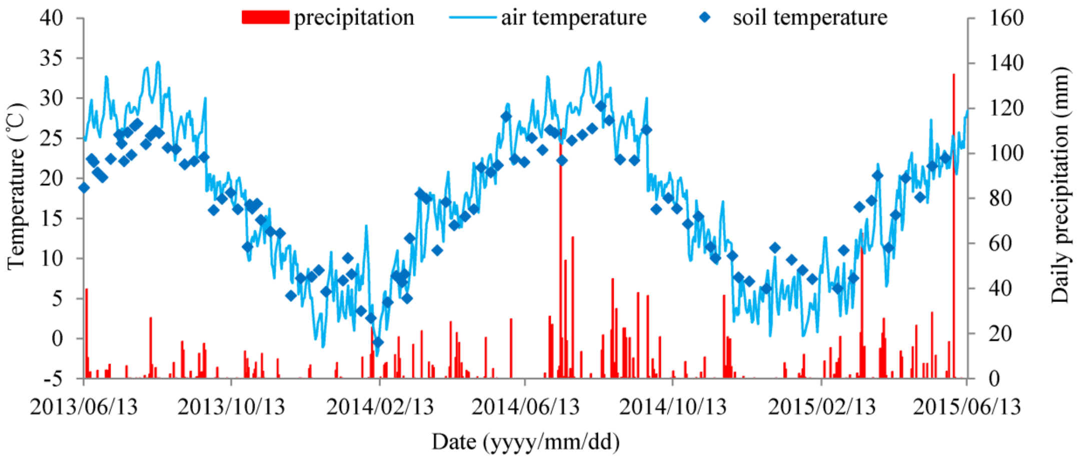

Fig. S3. Schematic design of the soil gas collection device

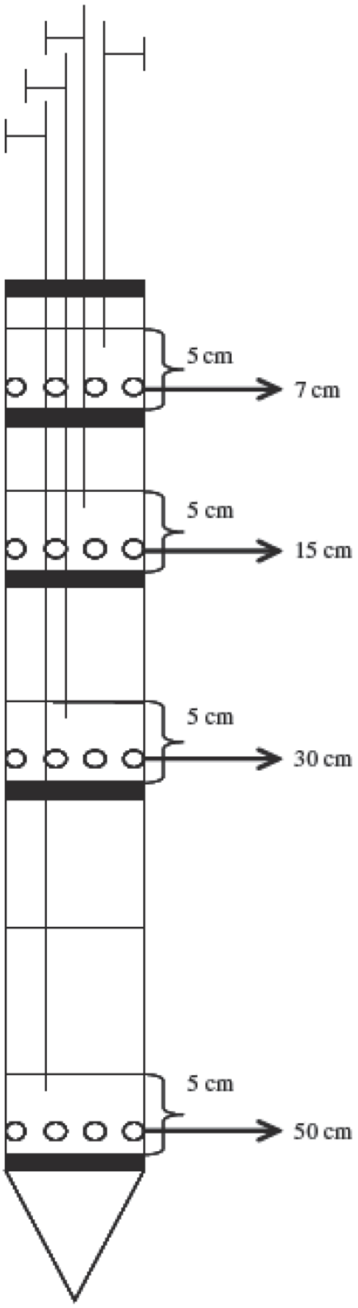

Table S1 Soil CH_4 concentration profiles (μL L^{-1}) among different N fertilizer and biochar treatments that were averaged over the rice and wheat seasons

Year	Crop season	Depth of soil (cm)	CH_4 concentrations (μL L^{-1})					Factors	*F*-value	*P*-value
			N0B0	N0B1	N1B0	N1B1	N1B2			
2013-2014	Rice season	7	5.31±1.76	7.22±0.57	27.83±3.74	25.84±6.05	17.92±1.26			
		15	5.56±1.27	7.44±0.71	20.29±1.82	16.35±3.82	14.39±1.20	Treatment	41.595	0.000
		30	5.71±1.58	6.43±0.50	13.61±1.01	10.10±3.52	10.35±1.81	Depth	46.91	0.000
		50	3.93±0.91	6.50±1.28	12.39±1.10	11.07±1.51	10.66±0.31	Treatment*Depth	9.151	0.000
	Wheat season	7	1.49±0.09	1.62±0.22	2.10±0.38	1.87±0.38	1.88±0.75			
		15	2.23±0.91	2.03±0.57	2.01±0.68	1.97±0.07	2.05±1.56	Treatment	1.207	0.367
		30	2.19±1.04	2.35±1.03	2.85±1.25	1.73±0.19	3.05±2.22	Depth	3.808	0.020
		50	2.72±1.39	2.92±0.55	3.13±1.32	1.39±0.51	2.23±0.84	Treatment*Depth	1.54	0.165
2014-2015	Rice season	7	5.08±1.48	6.46±1.15	39.53±9.56	28.92±7.23	19.87±2.41			
		15	4.15±0.10	4.77±1.67	24.92±4.20	19.23±3.34	12.60±3.66	Treatment	20.552	0.000
		30	4.69±0.67	4.47±0.85	21.63±2.88	14.30±7.04	15.83±1.99	Depth	16.598	0.000
		50	5.49±0.96	5.57±0.58	13.4 ±4.77	12.15±4.96	10.94±5.68	Treatment*Depth	3.852	0.001
	Wheat season	7	1.47±0.03	1.68±0.17	1.45±0.27	1.43±0.19	1.71±0.41			
		15	1.36±0.46	1.51±0.22	1.51±0.25	1.39±0.36	1.51±0.19	Treatment	1.491	0.277
		30	1.46±0.15	1.73±0.16	1.44±0.18	1.38±0.15	1.50±0.22	Depth	1.5	0.235
		50	1.60±0.12	1.88±0.44	1.81±0.17	1.36±0.13	1.39±0.30	Treatment*Depth	1.088	0.404

B0, B1 and B2 represent biochar applied at the rates of 0, 20 and 40 t ha^{-1}, respectively; N0 and N1 represent N fertilizer applied at the rates of 0 and 250 kg N ha^{-1} $crop^{-1}$, respectively.

Data are Means ± SD.

Table S2 The CH_4 emissions and diffusive effluxes (μg C m^{-2} h^{-1}) among different N fertilizer and biochar treatments averaged over the rice and wheat seasons

Year	Crop season	Δx/cm	CH$_4$ fluxes/mg C m^{-2} h^{-1}					Factors	*F* -value	*P*-value
			N0B0	N0B1	N1B0	N1B1	N1B2			
2013-2014	Rice season	Surface	1059.3±36.4	1056.6±31.2	2325.4±69.7	1859.5±70.5	1424.2±27.1			
		7–0	32.4±18.6	54.7±6.1	236.8±45.7	230.2±63.9	145.4±18.2	Treatment	5.686	0.001
		15–7	2.7±4.6	2.4±3.8	-50.9±32.1	-58.8±16.9	-19.1±4.9	Depth	194.791	0.000
		30–15	0.0±2.4	-4.4±2.7	-22.9±6.2	-19.7±13.2	-9.2±6.9	Treatment*Depth	20.256	0.000
		50–30	-4.0±1.6	0.5±3.0	-0.6±5.8	0.1±5.9	1.2±4.1			
	Wheat season	Surface	159.3±12.3	160.4±17.1	176.8±13.9	173.2±10.1	143.3±16.2			
		7–0	4.5±2.4	6.4±8.8	29.2±3.0	16.9±15.0	15.7±7.3	Treatment	0.527	0.716
		15–7	14.0±16.7	7.5±13.2	0.7±2.3	1.0±9.9	0.3±7.3	Depth	13.365	0.000
		30–15	-1.7±1.9	-1.1±5.5	-0.6±3.7	-1.5±1.4	3.0±6.1	Treatment*Depth	2.392	0.019
		50–30	1.1±1.4	2.6±1.1	0.0±1.6	-1.1±0.3	-2.0±4.2			
2014-2015	Rice season	Surface	334.3±49.9	553.8±138.4	1497.9±68.1	1367.3±83.0	1119.9±63.3			
		7–0	41.0±8.7	56.6±12.9	427.7±67.3	308.6±81.1	207.0±94.5	Treatment	4.737	0.003
		15–7	-7.0±9.1	-13.0±18.0	-111.5±41.0	-73.9±44.8	-55.5±22.7	Depth	117.530	0.000
		30–15	2.0±5.5	-1.1±8.6	-12.2±26.2	-18.3±13.8	-12.0±11.4	Treatment*Depth	15.341	0.000
		50–30	2.2±0.6	3.1±3.1	-23.0±18.9	6.0±32.9	-13.7±20.0			
	Wheat season	Surface	141.2±46.8	160.2±66.3	120.0±23.5	146.5±13.3	109.6±90.4			
		7–0	17.1±4.9	22.5±8.9	18.2±10.6	15.4±8.5	24.6±13.5	Treatment	0.507	0.731
		15–7	-2.0±3.3	-3.5±7.0	6.8±14.1	-2.6±5.7	-0.1±6.0	Depth	34.358	0.000
		30–15	0.5±1.1	2.7±1.2	-1.5±2.5	0.5±1.6	-1.7±2.0	Treatment*Depth	0.710	0.733
		50–30	-0.1±1.0	0.9±1.1	2.1±1.5	-0.2±1.6	0.4±2.3			

B0, B1 and B2 represent biochar applied at the rates of 0, 20 and 40 t ha^{-1}, respectively; N0 and N1 represent N fertilizer applied at the rates of 0 and 250 kg N ha^{-1} $crop^{-1}$, respectively.

Data are Means ± SD.

Table S3 The Basic physicochemical properties of soil in the different layers

Substances<br>Soil layer(cm)	pH<br>(1:2.5) H_2O	Organic C<br>(g kg^{-1})	Total N<br>(g kg^{-1})	CEC<br>(cmol kg^{-1})	Bulk density<br>(g cm^{-3})
0-7	5.91±0.16c	16.23±0.83a	1.43±0.07a	28.60±0.11a	1.15±0.03c
7-15	6.53±0.12b	12.51±1.30b	1.35±0.09ab	26.73±0.09a	1.20±0.02c
15-30	7.01±0.15a	8.72±1.41c	1.19±0.09bc	22.31±0.13bc	1.32±0.01b
30-50	6.72±0.20ab	6.91±1.07c	1.05±0.11c	19.80±0.11c	1.41±0.01a

Means ± SD, different lowercase letters within the same crop season in the same column indicate a significant difference at $P < 0.05$ based on Student's multiple range tests.

Table S4 Timetables of cultivation during the rice-wheat annual rotation system

Year	2013-2014		2014-2015	
Management measures	Rice season	Wheat season	Rice season	Wheat season
ploughing	2013/6/13	2013/10/28	2014/6/3	2014/11/3
Water flooding	2013/6/18	-	2014/6/9	-
Basal fertilizer applied	2013/6/19	2013/11/3	2014/6/10	2014/11/13
Sowing or transplanting	2013/6/19	2013/11/5	2014/6/11	2014/11/15
The first top-dressing	2013/7/12	2014/1/20	2014/7/7	2015/1/23
Mid-season drainage start	2013/7/19	-	2014/7/21	-
Mid-season drainage end	2013/7/25	-	2014/7/28	-
The second top-dressing	2013/8/6	2014/3/3	2014/8/12	2015/3/10
Gain	2013/10/18	2014/5/28	2014/10/25	2015/5/26
